# Novel health system strategies for tuberculin skin testing at primary care clinics: Performance assessment and health economic evaluation

**DOI:** 10.1371/journal.pone.0246523

**Published:** 2021-02-17

**Authors:** Eva Van Ginderdeuren, Jean Bassett, Colleen F. Hanrahan, Lillian Mutunga, Annelies Van Rie

**Affiliations:** 1 Witkoppen Clinic, Johannesburg, South Africa; 2 Family Medicine and Population Health, Faculty of Medicine and Health Sciences, University of Antwerp, Antwerp, Belgium; 3 Epidemiology, Johns Hopkins Bloomberg School of Public Health, Baltimore, Maryland, United States of America; McGill University, CANADA

## Abstract

**Background:**

Tuberculin skin test (TST) for guiding initiation of tuberculosis preventive therapy poses major challenges in high tuberculosis burden settings.

**Methods:**

At a primary care clinic in Johannesburg, South Africa, 278 HIV-positive adults self-read their TST by reporting if they felt a bump (any induration) at the TST placement site. TST reading (in mm) was fast-tracked to reduce patient wait time and task-shifted to delegate tasks to lower cadre healthcare workers, and result was compared to TST reading by high cadre research staff. TST reading and placement cost to the health system and patients were estimated. Simulations of health system costs were performed for 5 countries (USA, Germany, Brazil, India, Russia) to evaluate generalizability.

**Results:**

Almost all participants (269 of 278, 97%) correctly self-identified the presence or absence of any induration [sensitivity 89% (95% CI 80,95) and specificity 99.5% (95% CI 97,100)]. For detection of a positive TST (induration ≥ 5mm), sensitivity was 90% (95% CI 81,96) and specificity 99% (95% CI 97,100). TST reading agreement between low and high cadre staff was high (kappa 0.97, 95% CI 0.94, 1.00). Total TST cost was 2066 I$ (95% UI 594, 5243) per 100 patients, 87% (95% UI 53, 95) of which were patient costs. Combining fast-track and task-shifting, reduced total costs to 1736 I$ (95% UI 497, 4300) per 100 patients, with 31% (95% UI 15, 42) saving in health system costs. Combining fast-tracking, task-shifting and self-reading, lowered the TST health system costs from 16% (95% UI 8, 26) in Russia to 40% (95% UI 18, 54) in the USA.

**Conclusion:**

A TST strategy where only patients with any self-read induration are asked to return for fast-tracked TST reading by lower cadre healthcare workers is a promising strategy that could be effective and cost-saving, but real-life cost-effectiveness should be further examined.

## Introduction

About one fourth of the world’s population is infected with *Mycobacterium tuberculosis (Mtb)*, of which about 5–10% will develop active tuberculosis (TB) disease [1]. The risk for progression to active TB disease is especially high in people living with HIV (PLHIV) for whom TB preventive treatment (TPT) effectively reduces the risk of progression from *Mtb* infection to TB disease by 35% (RR 0.65; 95% CI 0.51, 0.84) [2]. To reach the TB elimination targets by 2035, TPT for all PLHIV is critical [3–5]. In 2018, the UN High Level Meeting on TB Political Declaration committed to providing TPT to at least 30 million people by the year 2022, including 4 million children, 20 million household contacts, and 6 million PLHIV [6].

The effectiveness of TPT in PLHIV depends on their *Mtb* infection status. Isoniazid preventive therapy (IPT) reduces the risk of active TB by 52% in PLHIV with a positive tuberculin skin test (TST) (RR 0.48, 95% CI 0.29, 0.82) compared to 21% (RR 0.79, 95% CI 0.58, 1.08) in those with a negative TST [2]. In 2011, the WHO therefore recommended that a TST is performed where feasible [7]. In 2014, the South African Department of Health (DOH) also recommended TST-guided TPT guidelines to target TPT to those likely to benefit most [8]. TST placement and reading however pose major challenges in resource limited settings, including logistical (cold chain, stock control), patient (the cost and burden of two clinic visits within 72 hours), and human resources (intradermal injection requires higher trained cadre of healthcare worker) [8–10]. Together, these hurdles result in low uptake of TST-guided TPT as only half of those eligible was tested for *Mtb* infection in low resource countries according to a systematic review [11, 12].

In high HIV burden countries, task-shifting (lower cadres of health care personnel performing tasks traditionally performed by nurses or doctors) and fast-tracking (rapidly delivering health care services to reduce patient waiting time) of HIV care services have gained a lot of attention given the scarcity of high cadre healthcare workers (HCW) and the overburdened healthcare system [13, 14]. Fast-tracking and task-shifting of TST reading could also be a cost-effective approach as people presenting for TST reading could be fast-tracked (resulting in reduced patient costs), and the reading could be task-shifted to lower cadre healthcare workers (resulting in reduced health system costs) [15, 16]. In addition, patient TST self-reading, where only those who self-read their TST as positive would return for TST reading at a health facility, is another innovative approach that could further improve the cost-effectiveness of TST given that an estimated 75% of people living in low and middle income countries are TST negative [17].

To assess if a combination of TST self-reading, fast-tracking and task-shifting is promising as an effective and cost-saving strategy, we evaluated the accuracy of TST self-reading (any versus no induration) among people receiving HIV care, the accuracy of TST reading by lower cadre staff, and estimated the net cost reduction from a primary care health system and societal perspective of these novel approaches.

## Methods

### Study setting

The study was performed in Witkoppen clinic in Northern Johannesburg, South Africa, a country with over 300.000 new TB cases annually, including 177.000 people co-infected with HIV in 2018 [18]. The Witkoppen primary healthcare facility serves a poor, densely populated, urban informal settlement of approximately 12 km^2^ with an estimated population of 138,329 [19]. During the study period (March 2016 to August 2017), the clinic provided TST-guided isoniazid preventive therapy (IPT) to all clients receiving HIV care as per national guidelines [8].

For the high cadre clinic staff, a refresher course on TST placement and reading was given, while for the low cadre clinic staff, a practical training session on TST reading was given. Both trainings were based on the Center for Disease Control and Prevention (CDC) TST training video and the TST training guide of the New Jersey Medical School Global Tuberculosis Institute [20, 21].

### TST self-reading, fast-tracking and task-shifting: Study population and procedures

Adults (age ≥ 18 years) living with HIV in whom a TST was placed by high cadre HCWs (as required by law) for routine care purposes were approached for study enrollment at their return visit for TST reading. Participant characteristics were obtained at enrollment (language spoken, highest level of education, and employment status), extracted from the medical files (age, gender, pregnancy status, date of HIV diagnosis, date of antiretroviral therapy initiation), or electronic laboratory database (most recent CD4 count).

First, study participants were asked to self-read their TST by using the flat tip of their right index finger to firmly rub back and forth over the TST site on the left forearm to feel if there was “a bump” or “no bump” present. Next, trained lower cadre healthcare workers (nursing assistants) fast-tracked the participants, read and recorded the size of TST induration in mm, blinded to the participant’s self-reported TST reading. Finally, a high cadre study staff (physician) read and recorded the size of TST induration in mm, blinded to reading of the lower cadre health staff.

### Data collection for costing analysis

To determine the cost of TST implementation, we followed the WHO-CHOICE methodology and measured costs prospectively using an ingredients approach through a combination of budgetary review, interview of administrative staff, and direct observation [22]. We observed 25 randomly selected TST placements and 25 TST readings. We used time-motion methodology to determine the average time needed for TST placement and TST reading. TST readings were only observed under the task-shifting strategy. Patient-level costs were measured by patient interview using the Stop TB Partnership’s “Tool to Estimate Patients’ Costs” [23]. We interviewed 73 patients at a regular clinic visit and 27 participants at a fast-track TST reading visit to estimate patient costs related to time spent at the clinic, duration and costs of transport, lost wages, childcare, cost spent on food during the clinic visit, and out-of-pocket costs related to a TST side effect (itching, pain, blistering or other ailment). Clinic fees were not included, as public primary clinics do not routinely request a clinic fee.

### Statistical analysis of TST self-reading and task-shifting performance

Baseline characteristics of participants were described using proportions or medians (IQR). Results of TST self-reading are presented in binary (“bump” versus “no bump”) format, results of formal TST reading in mm of induration (continuous), as any induration (≥ 1 mm) or no induration (0 mm), and as TST positive (≥ 5 mm) or TST negative (< 5 mm) [24].

To assess the accuracy of TST reading by lower cadre clinic staff in the task-shifting strategy, we compared TST readings by lower and high cadre staff (physician) using the kappa statistic, intraclass correlation coefficient (ICC) estimates and their 95% confidence intervals (CI). To assess the accuracy of TST self-reading (any versus no induration), we estimated the proportion of any indurations (≥ 1 mm) and positive TSTs (≥ 5 mm) missed by TST self-reading (no bump) and calculated the sensitivity, specificity, negative predictive value (NPV), and positive predictive value (PPV) of TST self-reading to detect any induration (≥ 1 mm) and to detect a positive TST (≥ 5 mm) with the clinic staff results as reference standard [25]. To identify patient-level factors associated with incorrect TST self-reading, we performed bivariate logistic regression analyses. We included sociodemographic (age, gender, language, work, education) and clinical variables (pregnancy, new diagnosis of HIV (i.e. < 90 days from enrollment), ART status, CD4 count) and used stepwise backward selection in the multivariable regression model. The goodness-of-fit of the stepwise logistic regression model was assessed using the Hosmer-Lemeshow test.

### Costing analysis

We estimated the health system and patient costs at the primary care setting per 100 patients in whom a TST was placed, and per 100 patients returning for TST reading under the standard and novel strategies. Health system level costs included consumables (e.g. PPD, syringes, etc.), equipment (e.g. pen, ruler, refrigerator for PPD storage), human resources (e.g. staff time and salary), infrastructure (e.g. clinic space, maintenance, security, water and electricity), and TST training (e.g. time and salary required). Time horizon was set at 1 year, capital inputs with a useful life > 1 year were valued using an annualization factor. The annual number of patients eligible for TST assessment at the clinic was estimated at 1500 and used as the denominator for annual costs [22, 26]. Patient time lost was not directly incorporated into the costs, but indirectly through the spending of food, lost wages, cost related to family care or payments to others because of clinic visit. A t-test was used to compare the average patient costs between the routine clinic visit and fast-track TST reading visit and 95% confidence intervals (95% CI) were calculated. Costs were converted to international dollars (I$) using purchasing power parity (PPP) conversion factors (World bank). Probabilistic uncertainty analysis was performed using 10.000 Monte Carlo simulations to account for the parameter uncertainty [27]. Parameters values were randomly sampled from their distributions bounded by a range which was set by the 95% CI for observed costs, by a published uncertainty range, or by a range of 50% if no data was available. The log-normal distribution was applied for cost parameters and the beta distribution for probabilities. The 95% uncertainty intervals (95% UI) were calculated as the 2.5^th^ and 97.5^th^ percentiles of those simulations.

To calculate the cost-reduction of the novel TST strategy from a health system perspective and a societal perspective (i.e. health system and patient costs), the cost of the standard of care approach (TST reading by high cadre HCW) was compared to that of 1) a task-shifting strategy (TST reading by low cadre HCW), 2) a fast-track strategy (fast-tracking patients for TST reading), or 3) a self-reading strategy (TST reading in patients who self-read TST as positive), and 4) the combination of these components.

To evaluate the generalizability of our findings, we simulated the costs from a health system perspective for 5 countries (USA, Germany, Brazil, India and Russia) using published data on key TST cost drivers (personnel cost and prevalence of *Mtb* infection) [17, 28–30]. These countries were selected to represent a range in income (lower and upper middle- or high-income countries), personnel costs (salary ratio between high and lower cadre from 1.6 to 6.6), and burden of *Mtb* infection among PLHIV (9 to 26%). For each setting, personnel costs used were hourly wages of lower cadres of HCWs (healthcare assistants) and higher cadres of HCWs (average of physicians and professional nurse combined). Personnel costs were converted to international dollars (I$) using PPP conversion factors (World bank) and inflated from study year (2011) to target year (2016) using the World bank country-specific consumer price index (CPI) deflator. Following the same methodology described above, the country specific estimates of burden of *Mtb* infection and HCW wages were then used to estimate the health system cost (and uncertainty interval) of the different TST strategies.

All analyses were performed in R version 3.6.1.

### Ethics approval

Ethical approval was obtained from the Human Research Ethics Committee of the University of the Witwatersrand (M150782). All participants gave written informed consent.

## Results

### Performance of TST self-reading and task-shifted TST reading

Of the 287 PLHIV approached and enrolled between March 2016 and August 2017, 278 had both TST self-reading and formal TST reading results. Of the 278 participants included in the analysis, median age was 34 years, 38% were unemployed, 79% did not complete secondary school and 71% were female, of whom 43 (22%) were pregnant (Table 1). Most (n = 200, 72%) had been diagnosed with HIV less than 90 days prior to study enrollment, and median CD4 count was 305 cells/mm^3^ (IQR 165–476).

**Table 1:** Sociodemographic and clinical characteristics of 278 study participants enrolled for self-reading.

		N = 278
**Sociodemographic**		**N (%)**
**Median age in years (IQR)**		34 (29–41)
**Gender**	Male	80 (29%)
	Female	198 (71%)
**Mother tongue**	National South African language	258 (93%)
	Foreign language	20 (7%)
**Education**	Secondary schooling or lower	220 (79%)
	Completed Matric	58 (21%)
**Employment status**	Unemployed	105 (38%)
	Employed or student	173 (62%)
**Clinical**		
**Pregnancy**	Pregnant	43 (15%)
	Not pregnant	235 (85%)
**HIV status**	Newly diagnosed (< 90 days)	200 (72%)
	Diagnosed > 90 days	78 (28%)
**ART status**	On ART	105 (38%)
	Not on ART	173 (62%)
**Median CD4 count in cells/mm^3^ (IQR)^¶^**		305 (165–476)

^¶^CD4 missing in 5 participants. IQR = interquartile range; ART = antiretroviral therapy.

Almost all (n = 269/278, 97%) participants correctly self-read their TST: 65 of 73 participants with TST induration ≥ 1 mm self-reported feeling a bump (89% sensitivity; 95% CI 80, 95), and 204 of 205 participants without a TST induration (0 mm) self-reported not feeling any bump (99.5% specificity; 95% CI 97, 100) (Table 2A). Among the 66 (24%) participants who felt an induration, 65 had an induration ≥ 1 mm, corresponding to a PPV of 98% (95% CI 92, 100) and NPV of 96% (95% CI 93, 98). Sociodemographic and clinical factors were not associated with TST self-reading errors (false negatives or positives) in logistic regression analysis.

**Table 2:** A) Performance of self-reading to detect any induration (≥ 1 mm). B) Performance of self-reading to identify a positive TST (induration of ≥ 5 mm).

A)				
	TST reading by routine clinic staff			
	Any induration (TST ≥ 1 mm)	No induration (TST = 0 mm)		
Patient feels a ‘bump’	65	1		PPV = 98%, 95% CI (92, 100)
Patient does not feel a ‘bump’	8	204		NPV = 96%, 95% CI (93, 98)
	Sensitivity = 89%, 95% CI (80, 95)	Specificity = 99.5%, 95% CI (97, 100)		
B)				
TST reading by routine clinic staff				
	Positive TST (TST ≥ 5 mm)		Negative TST (TST < 5 mm)	
Patient feels a ‘bump’	64		2	PPV = 97%, 95% CI (89, 100)
Patient does not feel a ‘bump’	7		205	NPV = 97%, 95% CI (93, 99)
	Sensitivity = 90%, 95% CI (81, 96)		Specificity = 99%, 95% CI (97, 100)	

TST = tuberculin skin test; CI = confidence interval; PPV = positive predictive value; NPV = negative predictive value.

Among the 73 (26%) participants with an induration ≥ 1 mm, the median size of induration was 16 mm (IQR 12–18, range 2–35) and 71 (26%, 95% CI 21,31) had a positive TST (induration ≥ 5 mm). Of these 71 participants, 7 (10%) failed to feel a bump. The sensitivity for the detection of a positive TST by TST self-reading was 90% (95% CI 81, 96), specificity 99% (95% CI 97, 100), PPV 97% (95% CI 89, 100), and NPV 97% (95% CI 93, 99) (Table 2B). Among those with a positive TST, participants with a smaller induration (5 to 10 mm) were more likely to self-report no induration compared to those with TST > 10 mm [4/12 (33%) participants with TST between 5–10 mm reported no induration vs 3/59 (5%) participants with TST > 10 mm, aOR 21.78 (95% CI 3.04, 242.43, adjusted for age and pregnancy)].

Overall agreement between TST reading by lower cadre clinic staff and high cadre study staff was excellent, with a kappa statistic of 0.97 (95% CI 0.94, 1.00, n = 257) and intraclass correlation coefficient of 0.97 (95% CI 0.97, 0.98). Among those with positive TST result (n = 67), intraclass correlation coefficient was 0.85 (95% CI 0.77, 0.91).

### Cost of standard and novel TST strategies at an urban clinic in Johannesburg, South Africa

The average time required to place a TST was 2.10 minutes (standard deviation (SD) = 0.40), to read a negative TST (< 5 mm) 1.74 minutes (SD 0.77), and to read a positive TST (≥ 5 mm) 2.88 minutes (SD 1.33). The duration of an annual TST training was 60 minutes. Units on consumables, equipment and infrastructure, and personnel for TST placement and reading, and their costs with 95% UI are detailed in Table 3. Parameters included in the probabilistic uncertainty analysis are detailed in Table 4. At Witkoppen clinic, the combined cost of personnel, consumables and equipment to place 100 TSTs (by higher cadre HCW) was 161 I$ (95% UI 125, 198). The health system cost for reading 100 TSTs in a population with a TST prevalence of 26% reduced from 110 I$ (95% UI 45, 195) using a standard strategy to 27 I$ (95% UI 13, 44) under a task-shifting strategy, assuming that all patients return for TST reading (Table 3). The majority of costs from a health system perspective were attributable to personnel costs (68% of TST placement costs and 87 to 97% of TST reading costs).

**Table 3:** Observed consumables, equipment and personnel costs (in I$) associated with TST placement and TST reading under a standard or a task-shifting TST strategy at a primary care clinic in urban South Africa.

	Units per patient	Cost (I$) per 100 patients^¶^	95% UI^£^	Proportion of total cost
**TST placement**				
**Infrastructure, equipment and consumables**				
Alcohol swab	1 unit	3.24		
Gloves	2 units	2.90		
Tuberculin PPD (RT23)	1 unit (2 TU)	17.32		
Syringe	1 unit	1.88		
Patient card	1 unit	12.29		
Refrigerator	Cost for 3 years^μ^^,^*	9.56	6.67, 16.82	
Cooler box (including cooling element)	Cost for 1 year*	1.75	1.22, 3.08	
TST register (1 copy)	0.2 unit^*#*^	1.71		
Use of 9 m^2^ room for HCW	1 room	0.44	0.27, 0.59	
Use of 60 m^2^ room for annual training	1 room*	0.06	0.04, 0.10	
Total		51.13	47.69, 59.74	*32% (26*, *41)*
**Personnel**				
Clinician staff	1 visit^&^	107.95	72.51, 142.63	
Training staff	1 annual training^&^^,^*	2.06	1.20, 3.83	
Total		110.01	73.88, 145.42	*68% (59*, *74)*
**Total health system cost for 100 TST placements**		**161.14**	**125.46, 197.70**	
**TST reading using a standard strategy~**				
**Infrastructure, equipment and consumables**				
Use of 9 m^2^ room by HCW	negative TST^&^	0.36	0.07, 0.68	
	positive TST^&^	0.60	0.12, 1.15	
Use of 60 m^2^ room for annual training	1 room^&^^,^*	0.04	0.04, 0.10	
Ruler	Cost for 1 year*	0.03	0.02, 0.04	
Pen	Cost for 1 month*	1.37	0.95, 2.40	
TST reading register (1 copy)	0.2 unit^*#*^	1.71		
Total	Overall^§^	3.57	3.07, 4.70	*3% (2*, *8)*
**Personnel**				
Healthcare worker	negative TST^&^	91.50	16.38, 179.56	
	positive TST^&^	150.10	27.84, 307.02	
Training staff	1 annual training^&^^,^*	2.06	1.20, 3.83	
Total	Overall^§^	106.74	41.77, 191.17	*97% (92*, *98)*
**Total health system cost for 100 TST readings using a standard scenario**^*§*^		**110.31**	***44*.*80*, *194*.*89***	
**TST reading using a task-shifting strategy~**				
**Infrastructure, equipment and consumables**				
Use of 9 m^2^ room by HCW	negative TST^&^	0.36	0.07, 0.68	
	positive TST^&^	0.60	0.12, 1.15	
Use of 60 m^2^ room for annual training	1 room^&^^,^*	0.04	0.04, 0.10	
Ruler	Cost for 1 year*	0.03	0.02, 0.04	
Pen	Cost for 1 month*	1.37	0.95, 2.40	
TST reading register (1 copy)	0.2 unit^*#*^	1.71		
Total	Overall^§^	3.57	3.07, 4.70	*13% (9*, *25)*
**Personnel**				
Healthcare worker	negative TST^&^	17.96	3.29, 36.05	
	positive TST^&^	29.73	5.59, 61.64	
Training staff	1 annual training^&^^,^*	2.06	1.20, 3.83	
Total	Overall^§^	23.07	7.99, 37.94	*87% (75*, *91)*
**Total health system cost for 100 TST readings using a task-shifting scenario**^*§*^		**26.65**	***13*.*48*, *44*.*08***	

^¶^Conversion to international dollars (I$) using PPP conversion factors (2016 World bank, PPP = 5.86)

^£^Using uncertainty ranges as described in Table 4

^μ^Annualization factor of 3% (2016 World bank) and 10% allocation factor for PPD storage

*Based on an annual number of 1500 patients eligible for TST assessment at the clinic and used as the denominator for annual costs

^#^Based on an average of 5 patients results registered per page;~Standard scenario is TST reading by higher cadre HCW while task-shifting scenario is TST reading by lower cadre HCW

^&^Based on observed average timings for TST placement (2.10 minutes), TST negative readings (1.73 minute), TST positive readings (2.88 minutes) and estimated annual training duration (60 minutes)

^§^Considering a 26% TST prevalence; TST = tuberculin skin test; I$ = international dollar; UI = uncertainty interval; PPD = purified protein derivative; TU = tuberculin units; HCW = healthcare worker.

**Table 4:** Base values and distributions of variables included in the probabilistic uncertainty analysis.

Variable	Base value	Distribution	Sources
TST positivity in PLHIV^#^	0.26 & Table 7^#^	Beta distribution bounded by lower and upper bound 95% CI (0.21, 0.31)	Observed; Ref [17, 28, 29]
Accuracy self-reading for detection positive TST			
• Sensitivity self-reading	0.90	Beta distribution bounded by lower and upper bound 95% CI (0.81, 0.96)	Observed
• Specificity self-reading	0.99	Beta distribution bounded by lower and upper bound 95% CI (0.97, 1.00)	Observed
The annual number of PLHIV eligible for TST	1500	Normal distribution bounded by range (500, 2500)	Observed; Ref [26]
Salary of high cadre and lower cadre HCWs	Table 3 and 7^#^	Normal distribution bounded by 50% range (50% of salary, 150% of salary)	Observed; Ref [30]
Timing of procedure			
• TST Placement	2.10 minutes	Normal distribution (mean = 2.10, SD = 0.40, min = 0 (truncated))	Observed
• Negative TST reading	1.73 minutes	Normal distribution (mean = 1.74, SD = 0.77, min = 0 (truncated)))	Observed
• Positive TST reading	2.88 minutes	Normal distribution (mean = 2.88, SD = 1.33, min = 0 (truncated)))	Observed
Patient cost for standard clinic visit	Table 5	Lognormal distribution (log-mean = 3.58; log-SD = 0.87)	Observed
Patient cost for fast-track clinic visit	Table 5	Lognormal distribution (log-mean = 3.36, log-SD = 0.62)	Observed

^*#*^See Table 7 for country-specific base values and ranges of TST prevalence and salaries of HCWs. TST = tuberculin skin test; PLHIV = people living with HIV; CI = confidence interval; HCWs = healthcare workers; SD = standard deviation.

The average waiting time for patients was 38 minutes (95% CI 19, 56) for a fast-track and 254 minutes (95% CI 223, 285) for a regular clinic visit. The longer waiting time resulted in more money spent on food while at the clinic (p = 0.005, Table 5). Among those employed, none (0/15) suffered wage loss due to a fast-track visit, 10% (or 5/48) lost wages due to a regular clinic visit (p = 0.03). Total average patient costs incurred were 8.97 I$ (95% CI 5.91, 12.03) for a standard clinic visit and 6.51 I$ (95% CI 3.30, 9.72, p = 0.26) for a fast-track visit, with transport to clinic and food spent while waiting at the clinic being the main patient costs.

**Table 5:** Observed patient costs (in I$) for standard clinic visit (TST placement) and fast-track clinic visit (TST reading) at a primary care clinic in urban South Africa.

	Standard clinic visit (n = 73)	Fast-track clinic visit (n = 27)
**Time lost (in minutes)**		
Average transport time to clinic (95% CI)	32 (29, 36)	32 (24, 40)
Average time in clinic (95% CI)	254 (223, 285)	38 (19, 56)
**Visit-related Costs (in I$**^**μ**^**)**		
Average transport cost to/from hospital (95% CI)	3.61 (3.30, 3.92)	3.60 (3.42, 3.79)
Average cost for family care (95% CI) (n = 99)	0.07 (0, 0.18)	0.63 (0, 1.53)
Average cost for food (95% CI)	2.33 (1.83, 2.84)	1.07 (0.37, 1.78)
Other (average payments to anybody that helped) (95% CI)	1.33 (0, 3.67)	1.20 (0, 3.41)
Average loss of wage (95% CI)	1.60 (0.12, 3.08)	0 (0, 0)
Average costs related to a TST side effect (95% CI)	NA	0 (0, 0)^#^
**Average total cost per patient (95% CI)**	**8.97 (5.91, 12.03)**	**6.51 (3.30, 9.72)**

^μ^Conversion to international dollars (I$) using PPP conversion factors (2016 World bank, PPP = 5.86). ^#^Six experienced a TST side effect. I$ = international dollar; TST = tuberculin skin test; CI = confidence interval.

The total cost (health system plus patient costs) associated with performing a TST under standard procedures at Witkoppen Clinic was 2066 I$ per 100 patients (95% UI 594, 5243) assuming that all patients return for TST reading (Table 6). Of this cost, 87% (95% UI 53, 95) was attributed to patient costs and 13% (95% UI 5, 47) to personnel, equipment and material costs. Introducing task-shifting reduced the total cost to 1982 I$ per 100 patients (95% UI 516, 5162). Introducing a fast-track strategy reduced the total cost to 1820 I$ per 100 patients (95% UI 576, 4399). Total costs for a strategy where self-reading is the only novel component introduced was 1332 I$ per 100 patients (95% UI 402, 3327). Self-reading plus task-shifting further reduced total costs to 1301 I$ per 100 patients (95% UI 372, 3299). Combining self-reading and fast-track strategies reduced total costs to 1268 I$ per 100 patients (95% UI 398, 3115). Employing both fast-tracking and task-shifting of TST reading resulted in overall program costs of 1736 I$ per 100 patients (95% UI 497, 4300). The lowest cost was achieved when employing a fast-track, task-shifting and TST self-reading strategy, with a total cost of 1237 I$ per 100 patients (95% UI 367, 3073), a saving of 40% (95% UI 35, 43) compared to the standard TST strategy.

**Table 6:** Estimated total cost for implementation of different TST strategies at a primary care clinic in urban South Africa.

	Definition of the TST strategy	Health system cost per 100 patients in I$ (95% UI)^μ^	Patient cost per 100 patients in I$ (95% UI)^μ^	Total cost per 100 patients in I$ (95% UI)	% reduction in total cost^*†*^ (95% UI)
**Standard TST strategy**^**#**^	TST reading by high cadre HCW	271 (185, 379)	1795 (322, 4971)	2066 (594, 5243)	Ref
**Task-shifting TST strategy**^**#**^	TST reading by low cadre HCW	188 (149, 230)	1795 (322, 4971)	1982 (516, 5162)	-4% (-1;-16)
**Fast-track TST strategy**^**#**^	Patient fast-tracked, TST reading by high cadre HCW	271 (185, 379)	1548 (307, 4107)	1820 (576, 4399)	-12% (-3;-16)
**Fast-track and task-shifting TST strategy**^**#**^	Patient fast-tracked, TST reading by low cadre HCW	188 (149, 230)	1548 (307, 4107)	1736 (497, 4300)	-16% (-11;-20)
**TST self-reading strategy**^£^	TST reading by high cadre HCW in patient who self-read TST as positive	201 (145, 262)	1131 (204, 3132)	1332 (402, 3327)	-36% (-30;-39)
**Task-shifting plus TST self-reading strategy**^£^	TST reading by low cadre HCW in patient who self-read TST as positive	170 (132, 205)	1131 (204, 3132)	1301 (372, 3299)	-37% (-33;-41)
**Fast-track TST strategy with TST self-reading**^£^	patient who self-read TST as positive TST, fast-tracked for TST reading by high cadre HCW	201 (145, 262)	1067 (200, 2911)	1268 (398, 3115)	-39% (-31;-42)
**Fast-track, task-shifting and TST self-reading strategy**^£^	patient who self-read TST as positive TST, fast-tracked for TST reading by low cadre HCW	170 (132, 205)	1067 (200, 2911)	1237 (367, 3073)	-40% (-35;-43)

^μ^Cost and 95% IU were calculated using item costs (see Table 3), base values and their distributions (see Table 4) and observed patient costs (see Table 5)

^†^% reduction in total cost of novel strategies compared to standard TST strategy

^**#**^assuming all patients return to clinic for TST reading

^£^assuming all patients with positive self-reading TST return to the clinic for confirmatory TST reading; I$ = international dollar; UI = uncertainty interval; TST = tuberculin skin test; HCW = healthcare worker.

### Simulation of health system cost of standard and novel TST strategies in 5 countries

We simulated the health system costs of different TST strategies in 5 countries to assess the impact of *Mtb* infection rates and HCW salaries (Table 7). Task-shifting for TST reading lowered the health system reading costs by 35% in countries with a low salary ratio (< 2 times difference in salaries between high and lower cadres of HCW) and by 75% in countries where the salaries of higher cadre HCW was over 4 times that of lower cadre HCW. A self-reading strategy lowered health system reading costs around 65% to 70% in countries with a high burden of *Mtb* infection (≥ 20%) and over 80% in countries with a lower burden of *Mtb* infection (< 20%). Compared to the standard TST strategy, employing a combined task-shifting and self-reading approach in a country with a high salary ratio (4.5) and low *Mtb* infection burden (9%) like the USA, reduced TST reading costs by 97% (95% UI 91, 99) and the total health system cost of the TST program (placement and reading) by 40% (95% UI 18, 54). In Russia, a country with a low salary ratio (1.8) and higher *Mtb* infection burden (26%), the reading costs reduced by 78% (95 UI 66,92) and total health system costs reduced by 16% (95% UI 8, 26). In countries like Germany with a low salary ratio (1.6) and low *Mtb* infection burden (12%) or countries like Brazil and India with higher salary ratios (6.6 and 3.2, respectively) and high *Mtb* infection burden (20% and 21%, respectively), reading costs reduced by about 90% and the total health system costs reduced by about 30%.

**Table 7:** Simulation of TST placement, reading and total TST costs in I$ per 100 patients (and 95% UI) from a health system perspective for 5 countries for a standard of care TST strategy, a task-shifting strategy, a self-reading strategy and a combined task-shifting and self-reading strategy.

	**USA**		**Germany**		**Brazil**		**India**		**Russia**		**South Africa~**	
Burden of *Mtb* infection among PLHIV (% and 95% CI)^&^	9 (6, 14)		12 (9, 16)		20 (17, 22)		21 (18, 24)		26 (20, 34)		26 (21, 31)	
Hourly salary high cadre (I$)^#^	50.21		20.75		13.84		11.95		3.84		30.84	
Hourly salary lower cadre (I$)^#^	11.24		12.93		2.09		3.69		2.13		6.19	
Salary ratio (high/lower cadre)^#^	4.5		1.6		6.6		3.2		1.8		5.0	
**TST placement health system cost in I$ per 100 patients (95% UI)**^*μ*^												
	**USA**		**Germany**		**Brazil**		**India**		**Russia**		**South Africa~**	
TST placement cost (95% UI)	**230** (171,290)		**125** (101,151)		**100** (84,119)		**94** (79,110)		**65** (59,75)		**161** (125, 198)	
**TST reading health system cost in I$ per 100 patients and % reduction of novel strategies compared to standard procedures (95% UI)**^*μ*^												
	**USA**		**Germany**		**Brazil**		**India**		**Russia**		**South Africa~**	
Standard TST strategy^#^^,^*	**161** (52,304)		**70** (25,126)		**50** (20,88)		**44** (18,76)		**17** (9,28)		**110** (45, 195)	
Task-shifting TST strategy^#^^,^*	**41** (17,74)	**-74%** (-59%;-83%)	**45** (18,80)	**-35%** (0;-58%)	**11** (7,18)	**-77%** (-63%;-84%)	**17** (9,27)	**-62%** (-42%;-74%)	**11** (6,17)	**-34%** (-7%;-54%)	**27** (13, 44)	**-76%** (-62%;-84%)
TST self-reading strategy^£^^,^*	**22** (4,51)	**-86%** (-56%;-97%)	**13** (3,26)	**-82%** (-52%;-96%)	**14** (3,27)	**-71%** (-42%;-92%)	**13** (3,25)	**-70%** (-41%;-92%)	**6** (2,11)	**-65%** (-43%;-88%)	**40** (8,77)	**-64%** (-33%;-91%)
Task-shifting and TST self-reading strategy^£^^,^*	**5** (2,7)	**-97%** (-91%;-99%)	**8** (3,10)	**-88%** (-75%;-96%)	**3** (2,3)	**-94%** (-89%;-98%)	**5** (2,5)	**-89%** (-80%;-96%)	**4** (2,4)	**-78%** (-66%;-92%)	**9** (4,10)	**-92%** (-84%,-97%)
**TST placement + reading health system cost in I$ per 100 patients and % reduction of novel strategies compared to standard procedures (95% UI)**^*μ*^												
	**USA**		**Germany**		**Brazil**		**India**		**Russia**		**South Africa~**	
Standard TST strategy^#^^,^*	**391** (253,573)		**195** (139,267)		**150** (112,200)		**138** (104,181)		**82** (70,99)		**271** (185, 379)	
Task-shifting TST strategy^#^^,^*	**272** (207,341)	**-31%** (-13%;-43%)	**171** (133,214)	**-13%** (0%;-24%)	**112** (94,132)	**-26%** (-11%;-37%)	**110** (93,131)	**-20%** (-8%;-30%)	**76** (68,88)	**-7%** (-1%;-14%)	**188** (149, 230)	**-31%** (-15%;-42%)
TST self-reading strategy^£^^,^*	**253** (184,326)	**-35%** (-12%;-50%)	**138** (108,170)	**-29%** (-10%;-43%)	**115** (92,140)	**-24%** (-8%;-36%)	**107** (87,129)	**-22%** (-8%;-35%)	**71** (63,82)	**-14%** (-6%;-22%)	**201** (145, 262)	**-26%** (-9%;-40%)
Task-shifting and TST self-reading strategy^£^^,^*	**236** (175,295)	**-40%** (-18%;-54%)	**133** (107,157)	**-32%** (-13%;-46%)	**103** (86,121)	**-31%** (-16%;-44%)	**98** (83,114)	**-28%** (-14%;-41%)	**69** (62,78)	**-16%** (-8%;-26%)	**170** (132, 205)	**-37%** (-19%;-51%)

^&^TST prevalence and 95% CI obtained or calculated from published data [17, 28, 29]

^#^Lower cadre healthcare workers (HCW) refers to personal healthcare assistants, high cadre HCWs to the average salary of medical doctors and professional nurses combined [30]

^μ^95% UI estimated according to uncertainty ranges as in Table 4

*see Table 6 for definition of the different strategies

^#^assuming all patients return to clinic for TST reading

^£^assuming all patients who self-read their TST as positive return to the clinic;~represent the non-simulated estimates of our study for comparison. TST = tuberculin skin test; I$ = international dollar; UI = uncertainty interval; Mtb = Mycobacterium tuberculosis; PLHIV = people living with HIV; HCW = healthcare worker.

## Discussion

At an urban primary care clinic in South Africa, we demonstrated that an innovative self-reading, fast-tracking and task-shifting TST strategy was highly performant for TST reading and greatly reduced patient and health system costs needed for a TST program from 2066 I$ to 1237 I$ per 100 PLHIV.

TST self-reading using a “bump” or “no bump” approach performed well among PLHIV, with a sensitivity of 90% and specificity of 99% for detection of a positive TST (≥ 5 mm). Results of prior TST-self-reading studies have been mixed, likely due to differences in populations and methods used. Poor performance was observed at a health promotion clinic in Seattle and in a population of PLHIV and drug users in New York [31, 32], while good performance was noted at an occupational health program for firefighters and among university students in the USA [33, 34]. In the only study of TST self-reading in a high TB/HIV burden country, 20% of the PLHIV’ self-read results were incorrect (compared to 3% in our study) but self-reading was recorded as positive (≥ 5 mm) or negative (< 5 mm) using a hole punched in a card whereas we used an easier “no bump” vs “any bump” approach [35].

Many overburdened primary care clinics have successfully implemented fast-tracking as a differentiated HIV care model and task-shifting to reduce costs by shifting tasks performed by nurses or doctors to lower cadres of healthcare personnel [13–16, 36]. In our study, we demonstrated that task-shifting of TST reading can be successfully implemented, without loss of reading accuracy (kappa 0.97) and observed that the fast-track system substantially reduced visit related patient costs.

Key cost drivers of TST programs were the prevalence of *Mtb* infection, the salary of healthcare workers and patient costs for clinic visits. In urban South Africa, a task-shifting and fast-tracking approach lowered the total TST costs (combined health system and patient perspective) per 100 patients by 16% from 2066 I$ to 1736 I$. Adding self-reading to the task-shifting and fast-tracking strategy further reduced the total cost by ~25%, to 1237 I$ per 100 PLHIV, as only those patients that feel an induration must come to the clinic for confirmatory TST reading.

Simulation of the impact on health system costs of the novel strategies in 5 countries demonstrated that the cost saving of the self-reading strategy was greatest in countries with low *Mtb* infection prevalence, as few patients need to return to the health facility for formal TST reading. Cost savings associated with a task-shifting approach were greatest in countries with high salary ratios between high cadre of healthcare workers (doctors and nurses) and lower cadre nursing assistants. Unfortunately, as data on patient costs were not readily available in the published literature, we could not assess the generalizability of the impact of these novel strategies on patient costs.

Our study has some limitations. First, in the absence of a gold standard, we assessed the performance of different implementation strategies of the TST to determine the *Mtb* infection status. Second, we performed the study among PLHIV in a single urban clinic in a South African setting limiting generalizability to other urban and rural clinics. To guide policy, our findings should be validated in other settings as costs may differ between urban and rural areas. Third, our simulation in low TB/HIV burden settings provided a first glance at generalization of our findings to those settings. A cost-analysis of real-life health system and patients costs of the novel TST strategies should be done to further validate these novel strategies. Fourth, we made a simplifying assumption that all patients returned to have their TST read. Future studies should assess the time and cost needed to train PLHIV to self-read their TST at home, the feasibility and acceptability of self-reading at home in particular the potential psychological consequences of being confronted with a positive test (i.e. diagnosis of TB infection) at home and the willingness to return, as well as the real-life proportion of PLHIV with a self-read positive induration that return to the clinic and all (indirect) costs associated with any loss of real-life effectiveness. Fourth, indirect costs such as time and wages lost were collected through self-report, which could introduce bias. Finally, we did not perform a formal cost-effectiveness analysis which will be required as next step to assess the real-life cost-effectiveness of the novel TST reading strategies.

**In conclusion,** a novel self-reading, fast-tracking and task-shifting model, which combines patient empowerment and differentiated care models, seems to be promising as an innovative, reliable and cost-saving approach to TST-guided tuberculosis preventive therapy for high and low TB burden settings but real-life cost-effectiveness in different settings should be examined.
